# A Toxicological Evaluation of a Standardized Hydrogenated Extract of Curcumin (CuroWhite™)

**DOI:** 10.1155/2018/5243617

**Published:** 2018-01-23

**Authors:** Alastimmanahalli Narasimhiah Ravikumar, Joby Jacob, Sreeraj Gopi, Tumkur Subbarao Jagannath

**Affiliations:** ^1^Liveon Biolabs (P) Ltd., Tumkur, Karnataka 572106, India; ^2^R&D Centre, Aurea Biolabs (P) Ltd., Kolenchery, Cochin, Kerala 682311, India

## Abstract

A series of toxicological investigations were conducted in order to evaluate the genotoxic potential and repeated-dose oral toxicity of CuroWhite, a proprietary extract of curcumin that has been hydrogenated and standardized to not less than 25% hydrogenated curcuminoid content. All tests were conducted in general accordance with internationally accepted standards. The test item was not mutagenic in the bacterial reverse mutation test or in vitro mammalian chromosomal aberration test, and no in vivo genotoxic activity was observed in rat bone marrow in the micronucleus test. A 90-day repeated-dose study was conducted in male and female Sprague-Dawley rats. Two mortalities occurred in the main and satellite high-dose groups and were determined due to gavage error. No organ specific or other toxic effects of the test item were observed up to the maximum dose of 800 mg/kg bw/day, administered by gavage. NOAEL was, therefore, estimated as 800 mg/kg bw/day.

## 1. Introduction

Curcuminoids, which are isolated from turmeric root (*Curcuma longa *Linn.), have a long history of use in the traditional Ayurvedic and Chinese medicines. Curcumin (C1), demethoxycurcumin (C2), and bisdemethoxycurcumin (C3) are the main components in curcumin and are responsible for its biological activities [1]. Curcumin and its structural analogues have many biological activities, such as cytoprotection, antioxidant activity, inflammatory response modification, cardiovascular support, neuroprotection, and radioprotection [2].

Catalytic hydrogenation of curcumin leads to tetrahydrocurcumin (THC), hexahydrocurcumin (HHC), and octahydrocurcumin (OHC) [3]. These are the major metabolites of curcumin; like their parent compounds, they have many biological activities [4, 5]. The effect of THC was studied against ferric nitrilotriacetate- (Fe-NTA-) induced oxidative stress in vivo [6]. THC is more easily absorbed from the gastrointestinal tract than curcumin [6] and induces antioxidant enzymes, such as glutathione peroxidase, glutathione S-transferase, and NADPH:quinone reductase, and scavenges Fe-NTA-induced free radicals in vitro better than curcumin. These results suggest that curcumin is converted to THC in vivo. Similarly, THC has a higher antioxidant activity than curcumin [7], and the antioxidant activity of THC has been analyzed for its effects on the oxidative modification of lipids in vitro. THC showed better antioxidative effects than alpha-tocopherol and curcumin [8]. THC supported normal vascular function in the presence of N*ω*-nitro-L-arginine methyl ester hydrochloride in rats, and the effects were associated with the alleviation of oxidative stress [9] while exposure of adenosine diphosphate treated human platelet-rich plasma to HHC resulted in an inhibitory effect on platelet aggregation [10]. These results suggest both compounds may have potential to support cardiovascular health. THC was investigated for its possible hepatoprotective effect compared with silymarin in Wistar rats against erythromycin estolate-induced toxicity [11]. The results of this study revealed that THC could afford significant protection compared to silymarin. Both curcumin and its metabolite THC exerted neuroprotection against 1-methyl-4-phenyl-1,2,3,6-tetrahydropyridine and inhibited the depletion of dopamine [12].

Hydrogenated curcuminoids have higher bioavailability while encapsulated with *β*-cyclodextrin compared with curcumin 95%. *β*-Cyclodextrin acts as a lipophilic cage and hence increases the aqueous solubility and stability of the active molecules [1]. CuroWhite is a unique formulation of hydrogenated curcuminoids encapsulated with -cyclodextrin. However, there are no studies available investigating the potential toxic effects of hydrogenated curcuminoids. Our research group conducted acute and subchronic oral toxicity studies of CuroWhite in rats and briefly summarized the results previously [13]. In the present work, we report the genotoxicity studies and provide detailed reporting of the previously summarized subchronic study of the hydrogenated curcuminoid formulation, CuroWhite.

## 2. Materials and Methods

### 2.1. Chemicals

All chemical reagents, solvents, pharmaceuticals, and other chemicals used in the studies were of analytical or pharmaceutical grade. Dimethylsulfoxide (DMSO) obtained from HiMedia Laboratories (Nasik, Maharashtra, India) was used in all three genotoxicity studies. Cyclophosphamide obtained from Sigma-Aldrich (St. Louis, MO, USA) was used in the in vitro mammalian chromosomal aberrations and in vivo mammalian micronucleus tests. The following additional chemicals were used in the bacterial reverse mutation and in vitro mammalian chromosomal aberration tests: D-glucose-6-phosphate, magnesium chloride, *β*-nicotinamide adenine dinucleotide phosphate monosodium salt, and potassium chloride (KCl) obtained from HiMedia Laboratories. The following additional chemicals were used in the bacterial reverse mutation test: 2-aminoanthracene (2AA), 2-nitrofluorene (2NF), 4-nitroquinoline 1-oxide (4NQO), 9-aminoacridine (9AA), and sodium azide (SAZ) obtained from Sigma-Aldrich and agar, ammonium sodium phosphate, citric acid, D-biotin, dextrose, dipotassium hydrogen phosphate, disodium hydrogen phosphate, L-histidine, L-tryptophan, magnesium sulfate, Oxoid nutrient broth number 2, potassium hydrogen phosphate, and sodium chloride obtained from HiMedia Laboratories. The following additional chemicals were used in the in vitro mammalian chromosomal aberration test: colchicine and mitomycin C obtained from Sigma-Aldrich; Dulbecco's phosphate buffer, fetal bovine serum (FBS), Giemsa stain, glutamine penicillin streptomycin solution, and Roswell Park Memorial Institute (RPMI) 1640 medium obtained from HiMedia Laboratories; and glacial acetic acid and methanol obtained from Fisher Scientific Co. (Mumbai, Maharashtra, India). May-Grünwald's stain obtained from HiMedia Laboratories was additionally used in the in vivo mammalian micronucleus test. The following chemicals were used in the 90-day oral toxicity study in rats: carbon dioxide (CO_2_) gas obtained from Sridevi Gas Agency (Tumakuru, India); DPX, K2-ethylenediaminetetraacetic acid, and formalin obtained from Nice Chemicals (Kochi, India); eosin obtained from reChem Laboratories (Canada); and haematoxylin obtained from Microexpress (Tumakuru, India).

### 2.2. Test Item

The test item was CuroWhite (Aurea Biolabs (P) Ltd., Kolenchery, Kerala, India). CuroWhite is manufactured in compliance with Good Manufacturing Practice by extraction of curcumin from turmeric (*Curcuma longa* L.) rhizome powder followed by hydrogenation, encapsulation with* beta*-cyclodextrin, and spray-drying to produce a 25–27% standardized hydrogenated curcumin powder with an off-white appearance [1]. Specifications for compositional analysis are shown in Table 1. Lot numbers SL162053 (genotoxicity studies) and SL151691, SL151719, and SL153146 (90-day oral toxicity study) were provided, together with specifications, certificates of analysis, and material safety data sheet, and the test item was identified in accordance with Good Laboratory Practice (GLP).

### 2.3. Genotoxicity Studies

#### 2.3.1. Bacterial Reverse Mutation Test

The bacterial reverse mutation test was conducted in general compliance with the Organisation for Economic Cooperation and Development (OECD) GLP as given in OECD C(97)186/Final [14] and in general accordance with OECD 471 test guideline [15] and* US FDA Redbook 2000*, IV.C.1.a [16] in order to investigate the mutagenic potential of CuroWhite. Bacterial tester strains* Salmonella *Typhimurium TA98, TA100, TA1535, and TA1537 and* Escherichia coli* WP2* uvrA* were obtained from MOLTOX Inc., P.O. Box 1189, BOONE, NC 28607 USA.

A preliminary solubility test was conducted by examining the test item mixed with distilled water, ethanol, acetone, glycerol, and DMSO. A precipitation test was conducted in duplicate with test item concentrations of 1–5 mg/plate and observation for 2 h at room temperature. Next, a preliminary cytotoxicity test was conducted in duplicate using tester strain* S. *Typhimurium TA100 with eight test item concentrations ranging from 0.05 to 5 mg/plate, with and without S9-mix.

Based on the preliminary cytotoxicity test results, seven concentrations (0.062, 0.185, 0.556, 1.667, 2.5, 3.75, and 5 mg/plate) were selected for the main mutagenicity assay. In all experiments, the test solutions were freshly prepared, just prior to treatment, and used within two hours by suspending the test item in DMSO at a concentration of 50 mg/mL and conducting serial dilutions to achieve the remaining concentrations, such that administration of 100 *μ*L of the test solutions achieved the above concentrations per plate. A cofactor supplemented postmitochondrial fraction (prepared in the laboratory from the liver extract of phenobarbital/*β*-naphthoflavone pretreated rats) metabolic activation system (S9-mix) was freshly prepared for use in all study phases just prior to treatment. Appropriate positive controls for use without S9-mix were chosen specific to the tester strain in accordance with the cited guidelines. The positive control with S9 was chosen based on in-laboratory characterization of the batch of S9 fraction used in the study with 2AA and benzo-(a)-pyrene. As both mutagens produced results within the historical positive control range of the laboratory, 2AA was chosen as the positive control for use with S9 with all tester strains. All positive controls were prepared with DMSO as the vehicle.

The main testing procedure consisted of both a standard plate incorporation (Method I) and preincubation (Method II) test. Each experiment of both methods was conducted in triplicate. Colony numbers were determined by counting; from these, mean values, standard deviations, and mutation rates were calculated. A result was considered positive if a concentration-related increase in revertant colonies occurred and/or a reproducible biologically relevant increase in revertant colonies for at least one concentration occurred in at least one strain with or without metabolic activation. Increases were considered biologically relevant if they fell above the upper confidence interval (95%) of the appropriate historical negative control. If neither of the above criteria was met, the test was considered negative.

#### 2.3.2. In Vitro Mammalian Chromosomal Aberration Test

The in vitro mammalian chromosomal aberration test was conducted in general compliance with OECD GLP [14] and in general accordance with OECD 473, 1997 test guideline [17] in order to investigate the clastogenic potential of CuroWhite. Two independent experiments were performed with and without S9 metabolic activation. Duplicate cultures (with and without metabolic activation) were maintained at each concentration of test item, solvent/vehicle-control, and positive control. The female Chinese hamster ovary cell line (CHO) used as the test system was obtained from American Type Culture Collection (USA, Manassas, Virginia) and grown in supplemented RPMI 1640 medium.

DMSO was used as the negative control and vehicle for the test item due to its compatibility with the test system. Test solutions were freshly prepared at the beginning of the range finding test, definitive assay, and confirmatory assay by dissolving the test item in DMSO at a concentration of 10 mg/mL and then conducting subsequent serial dilutions with RPMI 1640 medium to achieve the test solution concentrations for each experiment. The concentration of DMSO in RPMI for use as the negative control was 0.1%. The positive controls were prepared by dissolving the substances in DMSO to produce stock solutions, followed by serial dilutions with RPMI to achieve concentrations of 0.4 (definitive and confirmatory tests) and 0.8 (definitive test only) *μ*g/mL mitomycin C and concentrations of 7.5 and 15.0 *μ*g/mL cyclophosphamide (definitive test only). S9-mix was prepared in the laboratory as described in the Bacterial Reverse Mutation Test section.

A preliminary test was conducted to determine the solubility of the test item in DMSO, check for precipitation, and determine pH of the working stock solution. DMSO was added to 10 mg of the test item in 0.1 mL increments until it was fully dissolved, and the solubility concentration was calculated. The soluble stock solution was then serially diluted in RPMI 1640 medium to obtain a 0.1 mg/mL working stock solution, which was observed for precipitation and pH. A range finding cytotoxicity assay was conducted in duplicate following the same procedures used for the definitive assay experiments for the purpose of selecting concentrations for the main test. Cell counts were performed for calculation of mitotic indexes, and cytotoxicity was determined as relative cell growth (RCG) and relative mitotic index (RMI) (i.e., percent survival and percent dividing cells per 100 cells (based on scoring 1000 cells) in the treatment groups compared to the negative (solvent) control).

Two independent assays were conducted in the main test. In the definitive assay, CHO cultures were exposed to the negative or positive controls or test solution concentrations of 5, 10, and 15 *μ*g/mL (based on the range finding results) for a 3 h period with and without S9 metabolic activation. Following the exposure period, the cells were washed, supplemented with complete medium, and incubated for an additional 15 h. Sampling was made at 18 h (1.5 cell cycles) following the start of treatment.

The confirmatory assay was conducted as described for the definitive assay except that exposure to the test solution concentrations was for the entire 18 h incubation period and the experiment was conducted only without metabolic activation, due to negative results in the definitive assay. All individual test solutions and negative and positive control experiments were carried out in duplicate and concurrent measures of cytotoxicity were also conducted in the main experiments. Exposure and sampling times for definitive and confirmatory assays are summarized as follows:

- Definitive assay: 3 h treatment with and without S9-mix/18 h sampling time.
- Confirmatory assay: 18 h treatment without S9-mix/18 h sampling time.

Two hundred metaphase cells from each experimental group were evaluated for structural aberrations and scored. Polyploid and endoreduplicated cells were also recorded. The clastogenicity (i.e., negative or positive results) of the test item was determined as a concentration-related statistically significant increase in the frequency of chromosomal aberrations without gaps compared to the negative control and/or increases in the number of polyploid cells and endoreduplicated chromosomes compared to negative controls. The test item was considered nonmutagenic in the absence of the above criteria.

#### 2.3.3. In Vivo Mammalian Micronucleus Test

The in vivo mammalian micronucleus test was conducted in general compliance with OECD GLP [14] and in general accordance with OECD 474, 1997 test guideline [18] in order to investigate the in vivo genotoxic potential of CuroWhite. The test item doses were prepared by dissolving CuroWhite in DMSO to achieve concentrations of 20, 40, and 80 mg/mL in order to provide a constant dosing volume of 10 mL/kg bw. Dosing solutions were prepared daily and administered within three hours due to lack of stability data for the preparations. The negative control groups received the same volume of the DMSO vehicle only. The positive control was prepared by dissolving cyclophosphamide in saline to achieve a concentration of 5.0 mg/mL for administration of the standard dosing volume of 10 mL/kg bw.

Male and female Wistar rats (Liveon Biolabs, Karnataka, India) were utilized for the study and housed (maximum of 3 animals of the same sex/cage) in standard polypropylene cages with sterilized corncob for bedding at 22 ± 3°C, 42–68% relative humidity, and a 12 h light-dark cycle. The animals received AMRUT Laboratory Animal Feed (Pranav Agro Industries Ltd., Sangli, Maharashtra, India) and reverse osmosis purified water ad libitum. A preexperimental period of 6 days was provided to acclimatize the animals. Care and use of animals was in compliance with the recommendations of the Committee for the Purpose of Control and Supervision of Experiments on Animals (CPCSEA), Ministry of Environment, Forests, and Climate Change, Government of India, under the permission of the laboratory's Institutional Animal Ethics Committee (IAEC).

Fifty 6–8-week-old animals weighing 140–170 g were stratified by weight and randomly divided into groups of five rats/sex/group and given a single daily dose of the test item by gavage for two consecutive days at test concentrations of 0 (vehicle-control), 200, 400, and 800 mg/kg bw. The positive control, cyclophosphamide 50 mg/kg bw, was administered by gavage once.

Body weight measurements were obtained prior to the start of the experimental period and just prior to dosing on each study day. All animals were observed for mortality, visible signs of toxicity, or other reactions to treatment once daily (1 h after dosing) until sacrifice (by carbon dioxide exposure) 24 h following the final administration. Animals were subjected to gross necropsy and bone marrow smears were prepared in duplicate on standard microscope slides from samples obtained from the animals' femurs.

At least two thousand polychromatic erythrocytes (PCE) per animal were scored for frequency of micronuclei. The proportion of PCE to mature erythrocytes (a.k.a. normochromatic erythrocytes (NCE)) per animal was determined by the number of PCE and NCE encountered while counting at least 200 erythrocytes. The test result was considered positive if a statistically significant, dose-related increase, or increase in a single dose group, of micronucleated PCE (MPCE) was observed compared to controls. The result was considered negative if neither of the above two criteria was met.

### 2.4. Ninety-Day Repeated-Dose Oral Toxicity Study in Rats

The study was conducted in general compliance with GLP as given in OECD C(97)186/Final and in general accordance with OECD guideline 408 [19] in order to evaluate the potential health hazards associated with repeated oral exposure to CuroWhite and to estimate a no-observed-adverse-effect level (NOAEL). The study included two satellite groups for a 28-day, no-treatment, observational recovery period. Care and use of study animals was in accordance with CPCSEA guidelines and the laboratory's IAEC protocols.

One hundred male and female Sprague-Dawley rats (Liveon Biolabs (P) Ltd., Antharasanahalli, Karnataka, India), eight to nine weeks of age, weighing 140–169 g (males) and 140–170 g (females) at the start of the experimental period, were stratified by weight and randomly assigned to four main groups of 10 rats/sex/group and two satellite (recovery) groups of five rats/sex/group following a five-day (males) or six-day (females) acclimatization period. The animals (up to 3/cage) were housed in polypropylene cages with stainless steel mesh top grills and sterilized corncob bedding under conditions of adequate fresh air (12–15 changes/h), 21.2–24.8°C, 44–69% relative humidity, and a 12 h light-dark cycle. Laboratory animal feed (Pranav Argo Industries, Sangli, Maharashtra, India) and reverse osmosis well water were provided ad libitum.

CuroWhite was dissolved in distilled water (BML Industries, Bengaluru, India), as per the specification of the sponsor and its general acceptance as an appropriate vehicle for administration by gavage of water-soluble substances, in order to prepare the test item dosing formulations at concentrations of 20, 40, and 80 mg/mL. The test solutions were administered once daily by gavage at a constant dosing volume of 10 mL/kg bw (calculated weekly) to provide dose levels of 0 (vehicle-control), 200, 400, and 800 mg/kg bw/day as per the recommendation of the sponsor. The groups were designated as G1 (vehicle-control), G1R (vehicle-control recovery), G2 (low dose), G3 (middle dose), G4 (high dose), and G4R (high-dose recovery) as given in Table 2. Test solutions were freshly prepared each day and administered within three hours of preparation.

Clinical (morbidity and mortality, general cage-side, and detailed) and functional (reactivity to sensory stimuli, motor activity, and grip strength) observations, ophthalmological examinations, and measurements of body weight and feed consumption were made according to the OECD guideline, and body weight gain was calculated.

During the final week of the treatment (main groups) or the final week of the recovery period (satellite groups), urine samples were collected and examined for appearance, specific gravity, pH, protein, glucose, blood/blood cells, ketone bodies, nitrate, and leucocytes. Following the last treatment for the main groups or the last day of the recovery period for the satellite groups, and after an overnight fast, blood samples for measurement of clinical pathology parameters (i.e., hematology (including clotting time) and clinical chemistry) were obtained from all animals from the retroorbital plexus while under CO_2_ anesthesia. Clinical chemistry parameters measured deviated from the cited OECD guideline in that only two enzymes (alanine aminotransferase and aspartate aminotransferase) indicative of hepatocellular effects were measured.

Following blood collection, animals were euthanized by overexposure to CO_2_. Gross pathological examinations were performed, selected organ weights were measured, and organ weight to body weight ratios were calculated. Tissues from all animals were collected and preserved and full histopathological examinations (with the following deviations: tissues were not collected and examined from the pituitary and parathyroid glands) were conducted on those of the main control and high-dose groups. Full histopathological examinations were also conducted on two animals (one main high-dose male and one satellite high-dose female) found dead on Days 63 and 62, respectively.

### 2.5. Statistical Analyses

Microsoft Excel 2013 (Microsoft Corporation, Redmond, WA, USA) was used for statistical analysis of the chromosomal aberration test results utilizing the chi-square test for comparison of changes in numbers of cells with chromosomal aberrations compared to relevant controls. Statistical analyses were conducted using GraphPad Prism 5.04 for Windows (GraphPad Software, San Diego, CA, USA) for the mammalian micronucleus test and 90-day repeated-dose study. One-way analysis of variance (ANOVA) followed by Dunnett's posttest was conducted to evaluate changes in MCPE compared to controls in the mammalian micronucleus test. The same tests were conducted to compare significant differences between the main vehicle-control and treatment groups for body weight and body weight gain, feed consumption, absolute and relative organ weights, hematological and clinical chemistry parameters, and numerical urinalysis parameters in the 90-day study. These parameters were compared in the satellite control and high-dose group using Student's *t*-test. A *P* value of <0.05 was considered statistically significant.

## 3. Results and Discussion

### 3.1. Genotoxicity Studies

#### 3.1.1. Bacterial Reverse Mutation Test

In the preliminary solubility test, the test item was soluble in DMSO, forming a homogenous pale yellow suspension at a maximum concentration of 1000 mg/mL, but it was insoluble in the other tested solvents (data not shown). Therefore, DMSO was chosen as the vehicle and negative control for all experiments. No precipitation was observed at test item concentrations up to 5 mg/plate in the precipitation test, and no dose-related decreases in revertant colony numbers compared to the negative control or effects on background lawn development, with or without S9-mix, were observed at any test item concentration in tester strain TA100 in the preliminary cytotoxicity test (data not shown).

In Method I test (plate incorporation), mean revertant colony numbers, with and without metabolic activation, remained within the historical negative control range at all tested concentrations and in the concurrent negative control as shown in Table 3. In Method II test (preincubation), the concurrent negative control and test concentration of 0.185 mg/plate with S9-mix in tester strain TA98 fell slightly below the historical negative control range; however, as the differences were not statistically significant, these responses were considered normal. All other concentrations, with and without S9-mix, in all strains were within the historical range (Table 4). The positive controls in all experiments induced >2-fold increases in revertant colonies compared to concurrent negative controls, while no concentration-related or reproducible and biologically relevant increases in revertant colony numbers were observed in any strain at any test item concentration with or without metabolic activation in either test.

#### 3.1.2. In Vitro Mammalian Chromosomal Aberration Test

The test item was solubilized in DMSO at a concentration of 10 mg/mL, and no precipitation or change in pH was observed at the working stock concentration of 0.1 mg/mL following dilution of the vehicle stock solution in RPMI 1640 medium (data not shown). In the range finding test, at a concentration of 20 *μ*g/mL, RCG was reduced compared to the negative control by 76.18 and 67.56% in the absence and presence of S9-mix, respectively. Likewise, RMI was reduced by 55.71 and 57.89%, respectively, without and with metabolic activation. Due to the cytotoxicity observed at 20 *μ*g/mL, higher concentrations were not evaluated. Test item concentrations ≤ 10 *μ*g/mL resulted in reductions in RCG and RMIs < 35% of the negative control values (data not shown); therefore, a high concentration of 15 *μ*g/mL (midway between the lowest tested cytotoxic concentration and the highest tested noncytotoxic concentration) was chosen for use in the chromosomal aberrations test.

In the definitive assay, the percentage of negative control group cells with structural aberrations without gaps was 1.5% without and with S9-mix, and ≥12-fold increases in aberrant cells were observed in all positive control groups and were statistically significant compared to the negative controls and the results depicted in Table 5. No statistically significant differences were observed compared to negative controls in the percentages of chromosomal aberrations without gaps, at any test item concentration, with or without metabolic activation.

In the confirmatory assay (Table 6), conducted without S9-mix only, the percentage of negative control group cells with structural aberrations without gaps was 1.5%, and a 21-fold, statistically significant increase in aberrant cells was observed in the positive control. No statistically significant differences were observed compared to negative controls in the percentages of chromosomal aberrations without gaps, at any test item concentration. No polyploidy or endoreduplicated metaphases were observed in the test item-treated cells or negative controls, and no precipitation, effect on pH, or limiting cytotoxicity was observed under any of the experimental conditions of the definitive and confirmatory assays (data not shown).

#### 3.1.3. In Vivo Mouse Micronucleus Test

No mortality, clinical signs of toxicity, adverse reactions to treatment, or alterations in body weight and body weight gain were observed in any animals during the study (data not shown). The frequency of MPCE observed in the negative control group was within the historical control range of the laboratory, and a statistically significant increase in MPCE frequency was observed in the positive control group compared to negative control. No significant differences were observed in the frequency of MPCE between the three dose groups compared to the negative control as shown in Table 7. The proportion of PCE to mature erythrocytes was similar among the three dose groups and the negative control.

### 3.2. Ninety-Day Repeated-Dose Oral Toxicity Study in Rats

Two animals, a G4 (high-dose main group) male and a G4R (high-dose satellite group) female, were found dead on Days 63 and 62, respectively. No gross lesions were observed in the dead animals at necropsy. Bronchopneumonia was observed in the lungs of the G4 male, and congestion and edema of the alveoli as well as mild autolytic changes in the lungs and moderate to marked autolytic lesions in the liver, kidneys, stomach, intestine, genital organs, brain, sciatic nerve, spleen, thymus, and spinal cord were observed in both animals on microscopic examination (Table 9). No other mortalities were observed in the groups (G1 and G1R (vehicle-control), G2 (200), G3 (400), or G4 and G4R (800 mg/kg bw/day)) during the study.

Mild to moderate nasal discharge was observed in some animals of most treated groups beginning Week 1 in G3 and G4 males and G2 and G3 females, Week 3 in G4R males and G4 females, and Week 6 in G2 males and continuing transiently throughout the treatment period. In the high-dose satellite group (G4R) females, nasal discharge was observed on only a single occasion (Day 66) in a single animal, and no nasal discharge was observed at any time during the treatment or recovery periods in any of the control group (G1 and G1R) animals. No nasal discharge was observed in the G4R males after Week 10 or during the recovery period. A reduction in normal activity was observed transiently in a few individual animals of both sexes in all main treatment groups (one G1 male, Days 28–30; two G2 males, Days 13–15 and 49–56, resp.; one G4 male, Days 68–71; one each of G1 and G2 and two G4 females, Day 71; and one G4 female, Day 28), but it was not observed in any control or satellite group animals. No other clinical observations were observed in any other animals at the daily cage-side or weekly detailed observations throughout the treatment and recovery periods, and no abnormalities in responses to sensory stimuli, gait, or motor activity were observed in the main groups during the FOB.

No ophthalmological lesions or variations were observed in any G1 or G4 group animals prior to beginning or during the examination conducted the last week of treatment. No statistically significant variations in body weight, body weight gain, or feed consumption were observed during the treatment or recovery phases in any of the treated groups compared to the relevant controls (data not shown).

In the assessment of hematological parameters (data not shown), statistically significant increases in mean corpuscular hemoglobin concentration (MCHC) were observed in the G2 and G4 group females compared to controls. Despite being statistically significant, the increases in MCHC remained within the range of the laboratory's historical control data, and there was no statistically significant difference observed in MCHC of the G4R females compared to the satellite control at the end of the recovery period. No statistically significant differences compared to relevant controls were observed for any of the male treatment groups or in any other hematological parameters in the female groups.

A statistically significant dose-related decrease in creatinine compared to the main control group was observed in both sexes of the main groups on the clinical chemistry examination, while no statistically significant differences in creatinine were observed in the G4R group compared to G1R (Table 8). Several additional statistically significant alterations in clinical chemistry parameters compared to the relevant controls occurred sporadically and without a dose relationship among the groups and sexes. No statistically significant alterations were observed on the analyzed parameters of the urinalysis, and several sporadic alterations in blood, bilirubin, ketones, glucose, protein, and nitrates occurred among the groups and sexes with similar frequencies in control and treated animals or as individual findings absent of a dose relationship (data not shown).

On gross pathological examination at necropsy, no lesions were observed in any animals of any group. Statistically significant differences in absolute organ weights compared to relevant controls were observed only for increased liver weight in G4 males (data not shown). Several statistically significant increases in organ weights relative to body weight compared to the relevant controls were observed in the G4 male groups and occurred sporadically without clear dose relationships except in the case of liver weight to body weight ratio (Table 10). A statistically significant difference in relative organ weights was observed in the female groups only for a decreased liver weight relative to body weight in the G4R group compared to G1R (data not shown). All of the statistically significant alterations observed in absolute and relative organ weights were within the historical control range of the laboratory.

The microscopic lesions observed in the two animals that died during the study are reported above. Microscopic lesions observed in the main control and high-dose animals occurred with similar frequencies (or were greater in controls) or were isolated to individual animals. All microscopic lesions observed in the histopathological examination are shown in Table 9.

The genotoxic potential of CuroWhite, and to our knowledge of hydrogenated curcuminiods in general, has not been previously investigated. An in silico toxicity screening model predicted dihydrocurcumin, tetrahydro-bis-demethoxycurcumin, and tetrahydrodemethoxycurcumin (a synonym for demethoxytetrahydrocurcuminoid) to be potential mutagens and potential hepatotoxins, and dihydrocurcumin and tetrahydrodemethoxycurcumin were also predicted to be potential rodent carcinogens [20]. Of these hydrogenated curcuminoid compounds, only tetrahydrodemethoxycurcumin is a constituent of CuroWhite. Other hydrogenated curcuminoid constituents of CuroWhite do not appear to have been tested in these models, which is interesting in that tetrahydrocurcumin, hexahydrocurcumin, and octahydrocurcumin have been reported among the major hydrogenated curcuminoid metabolites of curcumin in several studies [21–25].

Curcumin has been fairly well studied and is generally recognized as safe for certain uses [26]; nonetheless, there is some disagreement regarding the carcinogenicity of curcumin. Curcumin (up to 500 and 160 *μ*g/plate) and curcumin oleoresin (up to 160 *μ*g/plate) have not shown mutagenicity in bacterial reverse mutation tests [27, 28], whereas curcumin induced chromosomal aberrations without metabolic activation in CHO cells at 10 *μ*g/mL [29, 30]. Curcumin has also been demonstrated to induce MPCE in human hepatoma G2 cells without metabolic activation [31]. However, curcumin nanoparticles, at doses up to 300 mg/kg bw, tested negative in in vivo chromosomal aberrations, micronucleus, and comet assays [32] and a curcuminoid-essential oil complex was negative in an in vivo chromosomal aberration test and an in vivo micronucleus test at doses of 2000 mg/kg bw [33]. Consistent with the above reported genotoxicity results, the National Toxicology Program (NTP) cited additional references demonstrating the lack of mutagenicity of curcumin in bacterial reverse mutation tests, in vitro induction of chromosomal aberrations and micronuclei, and the lack of in vivo chromosomal aberrations and micronuclei [34]. Therefore, due to mixed genotoxicity results and a general lack of oral toxicity and carcinogenicity tests, NTP conducted long-term carcinogenicity studies on curcumin in both rats and mice [34]. No evidence of carcinogenicity was observed in male rats following 2 years of ingestion of curcumin levels up to 2000 mg/kg bw/day in the diet, but evidence of curcumin-induced carcinogenicity was ruled equivocal in female rats and male and female mice by NTP based on increased incidence of several tumor types observed in various groups of animals in the studies without clear dose relationships. The Joint Food and Agriculture Organization of the United Nations/World Health Organization Expert Committee on Food Additives evaluated the NTP studies as part of its review of curcumin and concluded that curcumin is not a carcinogen because the observations were not dose-related [35]. Thus, because hydrogenated curcuminoids are normal metabolites of curcumin in humans, albeit at low levels [25], and because of the in silico predictions in other hydrogenated curcuminoids, we investigated the genotoxic potential of CuroWhite in the current work.

In the current bacterial reverse mutation test, in contrast to results observed by other groups with curcumin and curcumin oleoresin [27], no cytotoxicity was observed, and CuroWhite was evaluated up to the maximum recommended concentration of 5 mg/plate. Because the other acceptance criteria of no outlier numbers of spontaneous revertant colonies in the concurrent negative controls compared to the historical controls and appropriate mutagenic responses to the positive controls were fulfilled, the test was considered valid. Therefore, the results of both Method I and Method II, at concentrations of 0.062, 0.185, 0.556, 1.667, 2.5, 3.75, and 5 mg/plate, with and without metabolic activation, were considered unequivocally negative as all revertant colony numbers were far below genotoxicologically relevant thresholds.

In contrast with the works of Araujo et al. and others involving curcumin, CuroWhite did not induce statistically significant or concentration-related increases in structural chromosomal aberrations in CHO cells in either the definitive (3 h treatment with and without metabolic activation) or the confirmatory (18 h treatment without metabolic activation) assays of the current in vitro mammalian chromosomal aberrations test. Because the acceptance criteria for the positive and negative controls and cytotoxic concentrations were fulfilled, the test was considered valid, and the definitive and confirmatory assays without metabolic activation were considered unequivocally negative for clastogenicity.

The in vivo mammalian micronucleus test was considered valid as the assay acceptance criteria for negative and positive controls and proportion of immature among total erythrocytes were fulfilled. As no statistically significant increases in MPCE were observed compared to the negative control, the test was considered unequivocally negative. However, it is unclear whether bone marrow exposure to the test item occurred. While there was a slight depression in the P/E ratio in the female dose groups compared to the control group, this was clearly not the case in the male dose groups, and no sex differences in toxicity are expected as in Table 7. Thus, it is unclear whether the negative results observed should be interpreted as suggesting a lack of in vivo clastogenic activity of CuroWhite in rats. Nonetheless, based on the clearly negative results in the in vitro tests, as well as generally negative results of curcumin on in vivo micronucleus testing, genotoxicity of the test item is not expected.

The repeated-dose oral toxicity of CuroWhite was also investigated in the current work. In a previous acute oral toxicity study, CuroWhite was observed to be nontoxic at doses up to 2000 mg/kg bw in female Sprague-Dawley rats. These results were briefly summarized together with the results of the current 90-day study in a previous publication [13]. Because no other oral toxicity studies have been published on CuroWhite, or to the best of our knowledge hydrogenated curcuminoids in general, herein we described our 90-day study in detail.

The two deaths observed on study Days 62 and 63 (one high-dose recovery group female and one high-dose main group male) were considered accidental due to lung injury consistent with gavage error, the lack of other gross or histopathological findings that could suggest a toxic effect of the test item, and their isolated occurrence in individual animals (Table 9). In both animals, moderate to marked autolytic changes were observed in many organs and tissues and were considered due to the passage of time (estimated to be 12–16 h) between the rats dying during the night and their discovery at necropsy the following day.

Nasal discharge observed transiently in all treated groups throughout the treatment period was considered due to the strong pungent odor of the test item as it was not observed in control animals or in the G4R group during the recovery period. The only other clinical sign observed during the study was a reduction in activity occurring transiently in only a few animals of G1, G2, and G4 groups and was not considered test item-related due to its sporadic occurrence and absence in the G4R group at any time during the study.

No remarkable findings were observed in clinical pathology examinations with the exception of a statistically significant dose-related decrease in creatinine observed in both sexes (dose relation was evident in females while not as clearly evident in males, but it could not be ruled out) of the main groups compared to controls in the clinical chemistry evaluation (Table 8). While statistically significant, the decreases remained well within the historical control range of the laboratory and were not associated with any correlating histopathological observations, and no evidence of muscle wasting (or associated pathological conditions) was observed. Additionally, no evidence of conditions (e.g., liver disease, hemolytic anemia) that might result in a falsely lowered creatinine on blood analysis was evident. Finally, no statistically significant variations in creatinine were observed in the satellite high-dose group (G4R) compared to control (G1R). For these reasons, the alterations in creatinine were considered to have occurred without toxicological or biological relevance.

The increased liver weight observed at necropsy in G4 males was small in magnitude (remaining within the historical control range of the laboratory) and lacked a clear dose response, but it was associated with a dose-related statistically significant increase in liver weight relative to body weight in G4 males (Table 10); the latter finding was also within the historical control range, and both absolute and relative liver weights were recovered in the satellite group. No correlating findings were observed in the histopathological examination; thus, the observations were considered to be without toxicological relevance. No remarkable findings were observed in the gross or histopathological (except as described above for the animals found dead) examinations.

## 4. Conclusions

In conclusion, CuroWhite did not cause base-pair or frameshift mutations up to the maximum recommended concentration of 5 mg/plate in the bacterial reverse mutation test and was considered nonmutagenic under the applied conditions of the test system. Likewise, CuroWhite was considered nonclastogenic under the conditions of the in vitro mammalian chromosomal aberrations test as it failed to cause chromosomal damage up to the cytotoxic concentration of 15 *μ*g/mL. In the in vivo mammalian micronucleus test, no relevant increases in MPCE were observed up to 800 mg/kg bw and it was concluded that CuroWhite does not exhibit genotoxic activity under the applied conditions of the assay; however, it is noted that bone marrow exposure to the test item could not be unequivocally confirmed. In the 90-day oral toxicity study in male and female Sprague-Dawley rats, daily gavage administration of 0, 200, 400, and 800 mg/kg bw/day of CuroWhite did not cause toxic effects in the examined parameters, and NOAEL was estimated as 800 mg/kg bw/day. Because this was the highest dose tested, future studies utilizing higher doses and longer durations may be considered to further characterize the toxicological profile of CuroWhite.

## Figures and Tables

**Table 1:** Compositional specifications of CuroWhite.

Analyte/component	Result
	(% (w/w))
Total white curcuminoids	25–27
Tetrahydrocurcuminoids	16–22
Hexahydrocurcuminoids	1–6
Octahydrocurcuminoids	0.5–2
*β*-Cyclodextrin	≤75
Moisture content	<6
Total ash	≤0.5

**Table 2:** 90-day study dose groups.

Group	Treatment	Dose (mg/kg bw/day)	Treatment period (days)	Number of animals	
				Male	Female
G1	Vehicle-control	0	90	10	10
G2	Low dose	200		10	10
G3	Middle dose	400		10	10
G4	High dose	800		10	10
G1R	Vehicle-control recovery	0	90	5	5
G4R	High-dose recovery	800		5	5

**Table 3:** Summary of the results of Method I (plate incorporation test).

Test concentration (/plate)		Number of revertants/plate (mean of 3 plates)									
		Without S9					With S9				
		TA 98	TA 100	WP2 uvrA	TA 1537	TA 1535	TA 98	TA 100	WP2 uvrA	TA 1535	TA 1537

Historical negative control range		20–50	45–200	10–50	5–20	5–20	20–50	45–200	10–50	5–20	5–20
Solvent control (DMSO) (0.1 ml)	Mean	25	79	17	10	11	21	69	15	10	7
	SD	3	7	1	2	1	4	4	5	2	2
0.062 mg	Mean	27	70	16	9	9	23	78	18	9	7
	SD	4	10	4	2	2	4	6	3	2	2
0.185 mg	Mean	24	79	20	10	9	22	75	22	5	9
	SD	4	5	2	2	1	7	1	2	2	2
0.556 mg	Mean	26	81	25	8	9	25	79	21	8	11
	SD	3	5	4	3	3	2	3	2	2	2
1.667 mg	Mean	29	91	27	12	11	25	83	24	6	10
	SD	3	4	3	1	3	3	8	2	3	2
2.5 mg	Mean	28	84	28	11	10	22	81	28	8	7
	SD	3	2	2	2	3	4	6	2	2	3
3.75 mg	Mean	30	94	33	13	12	30	79	24	12	8
	SD	4	8	2	2	4	3	10	3	1	3
5 mg	Mean	34	98	30	12	15	29	85	26	9	11
	SD	2	1	6	4	2	1	6	4	2	3

		2-Nitrofluorene	Sodium azide	4-Nitroquinoline	9-Aminoacridine	Sodium azide	2-Aminoanthracene				

Positive controls	Mean	414	465	168	181	193	371	442	160	165	173
	SD	19	19	22	16	25	17	30	25	9	24

DMSO: dimethylsulfoxide; SD: standard deviation; TA:* Salmonella *Typhimurium tester strains; WP2 *uvr*A: *Escherichia coli* tester strain. *Notes*. All values are expressed as mean revertant colonies per plate ± standard deviation.

**Table 4:** Summary of the results of Method II (preincubation test).

Test concentration (/plate)		Number of revertants/plate (mean of 3 plates)									
		Without S9-mix					With S9-mix				
		TA 98	TA 100	WP2 uvrA	TA 1535	TA 1537	TA 98	TA 100	WP2 uvrA	TA 1535	TA 1537

Historical negative control range		20–50	45–200	10–50	5–20	5–20	20–50	45–200	10–50	5–20	5–20
Solvent control (DMSO) (0.1 ml)	Mean	20	68	43	11	12	17	65	32	10	9
	SD	4	3	3	4	3	2	5	3	3	2
0.062 mg	Mean	24	62	42	10	7	20	60	32	9	7
	SD	2	2	2	2	2	4	2	2	2	3
0.185 mg	Mean	28	76	44	15	8	19	81	40	13	11
	SD	3	2	4	2	3	3	1	2	4	2
0.556 mg	Mean	25	81	40	15	15	25	76	33	13	14
	SD	3	8	4	1	1	1	4	5	3	2
1.667 mg	Mean	23	83	44	14	15	25	78	34	14	12
	SD	5	3	3	4	4	4	1	3	3	3
2.5 g	Mean	23	78	43	16	14	26	74	31	12	16
	SD	6	2	3	4	3	5	3	2	3	3
3.75 mg	Mean	29	86	42	13	13	28	78	32	13	12
	SD	3	3	2	2	2	3	4	2	3	3
5 mg	Mean	33	80	35	14	17	25	82	33	13	14
	SD	4	4	4	4	2	1	3	5	3	2
		2-Nitrofluorene	Sodium azide	4-Nitroquinoline	Sodium azide	9-Aminoacridine	2-Aminoanthracene				

Positive controls	Mean	390	478	134	206	229	382	470	131	231	219
	SD	26	21	20	44	30	27	21	20	14	28

DMSO: dimethylsulfoxide; SD: standard deviation; TA:* Salmonella *Typhimurium tester strains; WP2 *uvr*A: *Escherichia coli* tester strain. * Notes. *All values are expressed as mean revertant colonies per plate ± standard deviation.

**Table 5:** Summary results of the definitive chromosomal aberration assay (treatment without and with S9-mix: 3 h, harvest: 18 h).

Test samples	Cytotoxicity test				Aberrations/100 cells					Number of cells with aberrations		% of cells with aberrations
	Relative cell growth (RCG)		Relative mitotic index (RMI)		Chromatid type		Chromosome type		Others			
	RCG%	Cytotoxicity%	RMI%	Cytotoxicity%	Gap+	Gap−	Gap+	Gap−		Gap+	Gap−	Gap−
*Without S9 activation*												
Negative control (DMSO, 0.1%)												
Parallel Slide 1	100	0	100	0	2	1	0	0	0	1	1	1.0
Parallel Slide 2					0	1	0	1	0	0	2	2.0
* Mean*					*1.0 *	*1.0 *	*0.0 *	*0.5 *	*0.0 *	*0.5 *	*1.5 *	*1.5*
Test item												
Parallel Slide 1 (5 *μ*g/mL)	95	5	72	28	1	2	0	0	0	1	2	2.0
Parallel Slide 2 (5 *μ*g/mL)					0	1	0	0	0	0	1	1.0
* Mean (5 μg/mL)*					*0.5 *	*1.5 *	*0.0 *	*0.0 *	*0.0 *	*0.5 *	*1.5 *	*1.5*
Parallel Slide 1 (10 *μ*g/mL)	82	18	70	30	0	4	0	1	0	0	3	3.0
Parallel Slide 2 (10 *μ*g/mL)					2	5	0	1	0	2	3	3.0
* Mean (10 μg/mL)*					*1.0 *	*4.5 *	*0.0 *	*1.0 *	*0.0 *	*1.0 *	*3.0 *	*3.0*
Parallel Slide 1 (15 *μ*g/mL)	66	34	56	44	0	11	0	3	0	0	6	6.0
Parallel Slide 2 (15 *μ*g/mL)					4	9	0	1	0	3	3	3.0
* Mean (15 μg/mL)*					*2.0 *	*10.0 *	*0.0 *	*2.0 *	*0.0 *	*1.5 *	*4.5 *	*4.5*
Positive control (mitomycin C)												
Parallel Slide 1 (0.4 *μ*g/mL)	40	60	84	16	1	15	0	5	1	1	19	19.0
Parallel Slide 2 (0.4 *μ*g/mL)					0	18	0	0	0	0	18	18.0
* Mean (0.4 μg/mL)*					*0.5 *	*16.5 *	*0.0 *	*2.5 *	*0.5 *	*0.5 *	*18.5 *	*18.5*
Parallel Slide 1 (0.8 *μ*g/mL)	24	76	53	47	3	28	0	10	1	3	23	23.0
Parallel Slide 2 (0.8 *μ*g/mL)					2	39	0	2	1	2	34	34.0
* Mean (0.8 μg/mL)*					*2.5 *	*33.5 *	*0.0 *	*6.0 *	*1.0 *	*2.5 *	*28.5 *	*28.5*
*With S9 activation*												
Negative control (DMSO, 0.1%)												
Parallel Slide 1	100	0	100	0	0	2	0	0	0	0	2	2.0
Parallel Slide 2					1	1	0	0	0	1	1	1.0
* Mean*					*0.5 *	*1.5 *	*0.0 *	*0.0 *	*0.0 *	*0.5 *	*1.5 *	*1.5*
Test item												
Parallel Slide 1 (5 *μ*g/mL)	86	14	70	30	0	2	0	0	0	0	1	1.0
Parallel Slide 2 (5 *μ*g/mL)					4	1	0	1	0	1	1	1.0
* Mean (5 μg/mL)*					*2.0 *	*1.5 *	*0.0 *	*0.5 *	*0.0 *	*0.5 *	*1.0 *	*1.0*
Parallel Slide 1 (10 *μ*g/mL)	69	31	59	41	5	0	0	0	0	1	0	0.0
Parallel Slide 2 (10 *μ*g/mL)					2	3	0	1	0	2	3	3.0
* Mean (10 μg/mL)*					*3.5 *	*1.5 *	*0.0 *	*0.5 *	*0.0 *	*1.5 *	*1.5 *	*1.5*
Parallel Slide 1 (15 *μ*g/mL)	53	47	56	44	1	8	0	1	0	1	7	7.0
Parallel Slide 2 (15 *μ*g/mL)					4	10	0	5	0	3	5	5.0
* Mean (15 μg/mL)*					*2.5 *	*9.0 *	*0.0 *	*3.0 *	*0.0 *	*2.0 *	*6.0 *	*6.0*
Positive control (cyclophosphamide)												
Parallel Slide 1 (7.5 *μ*g/mL)	44	56	28	72	2	38	0	8	1	2	33	33.0
Parallel Slide 2 (7.5 *μ*g/mL)					1	23	0	6	2	1	19	19.0
* Mean (7.5 μg/mL)*					*1.5 *	*30.5 *	*0.0 *	*7.0 *	*1.5 *	*1.5 *	*26.0 *	*26.0*
Parallel Slide 1 (15 *μ*g/mL)	39	61	21	79	4	52	0	11	2	4	50	50.0
Parallel Slide 2 (15 *μ*g/mL)					0	35	0	8	1	0	32	32.0
* Mean (15 μg/mL)*					*2.0 *	*43.5 *	*0.0 *	*9.5 *	*1.5 *	*2.0 *	*41.0 *	*41.0*

DMSO: dimethylsulfoxide.

**Table 6:** Summary results of the confirmatory chromosomal aberration assay (treatment without S9-mix: 18 h, harvest: 18 h).

Test samples	Cytotoxicity test				Aberrations/100 cells					Number of cells with aberrations		% of cells with aberrations
	Relative cell growth (RCG)		Relative mitotic index (RMI)		Chromatid type		Chromosome-type		Others			
	RCG%	Cytotoxicity%	RMI%	Cytotoxicity%	Gap+	Gap−	Gap+	Gap−		Gap+	Gap−	Gap−
*Without S9 activation*												
Negative control (DMSO, 0.1%)												
Parallel Slide 1	100	0	100	0	1	1	0	1	0	1	2	2.0
Parallel Slide 2					2	0	0	1	0	1	1	1.0
* Mean*					*1.5*	*0.5*	*0.0*	*1.0*	*0.0*	*1.0*	*1.5*	*1.5*
Test item												
Parallel Slide 1 (5 *μ*g/mL)	95	5	72	28	3	2	0	0	0	2	2	2.0
Parallel Slide 2 (5 *μ*g/mL)					1	0	0	2	0	1	2	2.0
* Mean (5 μg/mL)*					*2.0*	*1.0*	*0.0*	*1.0*	*0.0*	*1.5*	*2.0*	*2.0*
Parallel Slide 1 (10 *μ*g/mL)	82	18	70	30	3	2	0	2	0	2	4	4.0
Parallel Slide 2 (10 *μ*g/mL)					1	3	0	1	0	1	3	3.0
* Mean (10 μg/mL)*					*2.0*	*2.5*	*0.0*	*1.5*	*0.0*	*1.5*	*3.5*	*3.5*
Parallel Slide 1 (15 *μ*g/mL)	66	34	56	44	5	16	0	5	0	2	8	8.0
Parallel Slide 2 (15 *μ*g/mL)					3	8	0	3	0	2	5	5.0
* Mean (15 μg/mL)*					*4.0*	*12.0*	*0.0*	*4.0*	*0.0*	*2.0*	*6.5*	*6.5*
Positive control (mitomycin C)												
Parallel Slide 1 (0.4 *μ*g/mL)	24	76	35	65	5	33	1	16	3	6	25	25.0
Parallel Slide 2 (0.4 *μ*g/mL)					3	49	0	4	0	3	37	37.0
* Mean (0.4 μg/mL)*					*4.0*	*41.0*	*0.5*	*10.0*	*1.5*	*4.5*	*31.0*	*31.0*

DMSO: dimethylsulfoxide. *Notes.* Gap+ indicates scores including gaps. Gap− indicates scores excluding gaps. “Others” indicates polyploid cells and endoreduplicated metaphases. Statistical analysis was conducted on % aberrant cells using the chi-square test.

**Table 7:** Summary results of the micronucleus test.

Groups (*n* = 5)	Mean P/E ratio	Mean PCE analyzed	MPCE		
			Mean #	Mean%	SD
*Males*					
Vehicle-control	0.54	2093.8	19.8	0.95	±0.08
Cyclophosphamide (50 mg/kg bw)	0.52	2041.4	134.6	6.60^*∗∗∗*^	±0.76
200 mg/kg bw	0.55	2015.0	20.4	1.01	±0.05
400 mg/kg bw	0.53	2028.4	20.0	0.99	±0.11
800 mg/kg bw	0.56	2023.2	21.8	1.08	±0.07
*Females*					
Vehicle-control	0.54	2021.8	26.6	1.32	±0.65
Cyclophosphamide (50 mg/kg bw)	0.50	2017.0	129.6	6.43^*∗∗∗*^	±0.38
200 mg/kg bw	0.51	2014.6	20.2	1.00	±0.13
400 mg/kg bw	0.50	2021.6	22.8	1.13	±0.11
800 mg/kg bw	0.49	2011.0	24.6	1.22	±0.16

MPCE: micronucleated polychromatic erythrocytes; P/E: polychromatic erythrocytes/total erythrocytes; PCE: polychromatic erythrocytes; SD: standard deviation. *Symbols.  *^*∗∗∗*^*P* < 0.001. *Notes. *All values are expressed as means (% MPCE expressed as mean ± SD). Statistical analysis using one-way ANOVA followed by Dunnett's posttest, as compared to vehicle-control.

**Table 8:** Summary of statistically significant clinical chemistry parameters.

Group (mg/kg bw/day)	TPROT (g/dL)	Glucose (mg/dL)	ALT (IU/L)	TG (mg/dL)	Urea (mg/dL)	CREA (mg/dL)	Na^+^ (mmol/L)
*Main groups, male *(*n* = 10^†^)							
G1 (vehicle-control)	6.99 ± 0.68	66.38 ± 15.68	52.16 ± 12.56	62.38 ± 15.37	38.43 ± 8.11	0.79 ± 0.18	140.56 ± 6.16
G2 (200)	6.59 ± 0.50	65.31 ± 8.68	52.16 ± 10.32	56.29 ± 15.14	46.38^*∗*^ ± 9.03	0.64^*∗*^ ± 0.09	144.54 ± 5.83
G3 (400)	6.51 ± 0.77	94.39^*∗*^ ± 37.90	48.44 ± 6.63	40.75^*∗∗*^ ± 12.19	51.78^*∗∗∗*^ ± 5.31	0.64^*∗*^ ± 0.10	148.68^*∗∗*^ ± 5.85
G4 (800)	7.04 ± 0.65	85.37 ± 12.00	49.50 ± 15.18	57.67 ± 17.32	42.76 ± 2.40	0.60^*∗∗*^ ± 0.13	144.50 ± 5.27
*Recovery groups, male *(*n* = 5)							
G1R (vehicle-control)	8.65 ± 0.64	76.41 ± 14.62	50.92 ± 6.30	82.15 ± 10.62	46.24 ± 7.67	0.16 ± 0.05	155.04 ± 28.97
G4R (800)	8.49 ± 1.20	70.55 ± 8.57	65.06^*∗∗*^ ± 5.06	73.77 ± 27.87	53.96 ± 11.60	0.17 ± 0.03	124.56 ± 13.84
*Main groups, female *(*n* = 10)							
G1 (vehicle-control)	6.45 ± 1.22	68.00 ± 16.75	42.26 ± 6.48	55.35 ± 20.03	50.70 ± 6.90	0.75 ± 0.14	143.31 ± 10.40
G2 (200)	6.86 ± 0.78	68.91 ± 19.32	50.56 ± 9.94	52.66 ± 23.03	49.27 ± 6.61	0.69 ± 0.14	146.79 ± 6.17
G3 (400)	7.09 ± 1.00	59.61 ± 16.43	46.85 ± 10.68	37.27^*∗*^ ± 5.06	49.46 ± 7.14	0.64 ± 0.08	147.37 ± 9.87
G4 (800)	6.44 ± 0.37	65.16 ± 16.32	51.27 ± 8.04	41.17 ± 7.37	44.65 ± 5.62	0.60^*∗*^ ± 0.09	145.26 ± 7.69
*Recovery groups, female *(*n* = 5^‡^)							
G1R (vehicle-control)	8.44 ± 0.25	115.90 ± 20.89	40.67 ± 6.00	70.59 ± 23.91	53.89 ± 11.02	0.79 ± 0.20	192.54 ± 59.07
G4R (800)	7.93^*∗*^ ± 0.27	119.10 ± 34.09	47.74 ± 4.33	59.63 ± 42.00	42.21 ± 4.47	0.81 ± 0.08	143.50 ± 27.56

ALT: alanine aminotransferase; CREA: creatinine; Na^+^: sodium; TG: triglycerides; TPROT: total protein. *Symbols.  *^*∗*^*P* < 0.05; ^*∗∗*^*P* < 0.01; ^*∗∗∗*^*P* < 0.001; ^†^*n* = 9 in the G4 male group; ^‡^*n* = 4 in the G4R female group. *Notes.* All values are expressed as mean ± SD. Statistical analysis of main groups using one-way ANOVA followed by Dunnett's posttest as compared to vehicle-control. Statistical analysis of recovery groups using Student's *t*-test as compared to vehicle-control.

**Table 9:** Summary of histopathological findings.

Group (mg/kg bw/day)	G1-control (*n* = 10)	G4-800 (*n* = 10)	G4R-800 (*n* = 5)
*Organs and observations, male*			
Multiple organs: autolytic changes	0/10	1/10^†^	/
Liver: MNC infiltration	5/10	2/10	/
Kidneys: MNC infiltration	5/10	1/10	/
Lungs: MNC infiltration, perivascular	2/10	1/10	/
Lungs: bronchopneumonia, congestion, alveolar edema	0/10	1/10^†^	/
Salivary glands: MNC infiltration	2/10	0/10	/
Spinal cord: MNC infiltration	1/10	1/10	/
Epididymis: MNC infiltration	1/10	0/10	/
Eyes: periorbital inflammation	1/10	1/10	/
Urinary bladder: MNC infiltration	0/10	1/10	/
*Organs and observations, female*			
Multiple organs: autolytic changes	0/10	0/10	1/1^‡^
Liver: MNC infiltration	5/10	2/10	0/1
Kidneys: MNC infiltration	5/10	2/10	0/1
Lungs: MNC infiltration, perivascular	5/10	3/10	0/1
Lungs: congestion, alveolar edema	0/10	0/10	1/1^‡^
Thymus: epithelial cyst	1/10	0/10	0/1

MNC: mononuclear cell. *Symbols.  *^/^Not examined. ^†^Animal number 31 was found dead on Day 63. ^‡^Animal number 99 was found dead on Day 62. *Notes*. Data represent the incidence of the observation (number of animals with observation per number of animals observed).

**Table 10:** Summary of organ weights relative to body weight in male animals.

Group	Treatment and dose (mg/kg)	Fasting bw	Adrenals	Spleen	Thymus	Testes	Brain	Heart	Epididymis	Kidneys	Liver
*Main groups *(*n* = 10^†^)											
G1	Vehicle control	321.52 ± 47.57	0.02 ± 0.00	0.33 ± 0.07	0.10 ± 0.02	0.96 ± 0.11	0.57 ± 0.06	0.31 ± 0.02	0.40 ± 0.07	0.61 ± 0.04	2.65 ± 0.36
G2	Low dose-200	308.01 ± 24.27	0.02 ± 0.00	0.32 ± 0.06	0.09 ± 0.02	1.02 ± 0.08	0.59 ± 0.05	0.31 ± 0.03	0.42 ± 0.04	0.67 ± 0.05	2.98 ± 0.35
G3	Middle dose-400	310.90 ± 50.98	0.02 ± 0.00	0.33 ± 0.10	0.11 ± 0.04	0.98 ± 0.11	0.61 ± 0.08	0.34 ± 0.03	0.41 ± 0.07	0.67 ± 0.12	3.00 ± 0.46
G4	High dose-800	302.74 ± 28.47	0.02 ± 0.00	0.39 ± 0.14	0.10 ± 0.03	1.08^*∗*^ ± 0.12	0.57 ± 0.06	0.33 ± 0.02	0.42 ± 0.04	0.71^*∗*^ ± 0.07	3.54^*∗∗*^ ± 0.55
*Recovery groups *(*n* = 5)											
G1R	Vehicle control-recovery	334.42 ± 47.40	0.04 ± 0.05	0.30 ± 0.02	0.10 ± 0.01	1.00 ± 0.08	0.55 ± 0.11	0.35 ± 0.02	0.43 ± 0.04	0.61 ± 0.04	2.49 ± 0.15
G4R	High-dose recovery-800	325.18 ± 47.55	0.02 ± 0.00	0.28 ± 0.06	0.07 ± 0.02	0.97 ± 0.11	0.55 ± 0.06	0.32 ± 0.03	0.41 ± 0.03	0.59 ± 0.02	2.65 ± 0.37

*Symbols.* ^*∗*^ *P* < 0.05; ^*∗∗*^*P* < 0.01; ^†^*n* = 9 in the G4 male group. *Notes.* All values are expressed as mean ± SD. Statistical analysis of main groups using one-way ANOVA followed by Dunnett's posttest as compared to vehicle-control. Statistical analysis of recovery groups using Student's *t*-test as compared to vehicle-control.

## Additional Points

*Highlights*. (i) CuroWhite was not mutagenic in the bacterial reverse mutation test. (ii) CuroWhite exhibited no in vivo genotoxic activity. (iii) A NOAEL of 800 mg/kg bw/day—highest tested dose—was estimated in the 90-day study. (iv) No target organs or treatment-related toxicological effects were identified.
